# Supplementary material for: Voluntary Oral Ingestion of a Sedative Prior to Euthanasia with CO2: Behavioural Responses of Mice
Source: Animals (Basel). 2021 Oct 1;11(10):2879. doi: 10.3390/ani11102879 (PMC8533016; doi:10.3390/ani11102879)
Supplement: Supplementary file 1 [file animals-11-02879-s001.zip › animals-1366883-SI.pdf]

animals

Article

Voluntary Oral Ingestion of a Sedative Prior to Euthanasia with CO_2: Behavioural Responses of Mice

Raquel Rodriguez-Sanchez [1], Elyssa Barnaby [2], Lucia Améndola [3], Shen-Yan Hea [4], Bobby Smith [5], James Webster [2] and Gosia Zobel [1,*]

[1] Animal Behaviour and Welfare Team, AgResearch Ltd., Ruakura Research Centre, 10 Bisley Road, Private Bag 3123, 3214 Hamilton, New Zealand; Raquel.Rodriguez@agresearch.co.nz
[2] Animal Ethics Office, AgResearch Ltd., Ruakura Research Centre, 10 Bisley Road, Private Bag 3123, 3214 Hamilton, New Zealand; Elyssa.Barnaby@agresearch.co.nz (E.B.); jim.webster@agresearch.co.nz (J.W.)
[3] Atlantic Veterinary College, University of Prince Edward Island, 550 University Ave, Charlottetown, PE C1A 4P3, Canada; luciamendola@gmail.com
[4] Bioinformatics and Statistics, AgResearch Ltd., Grasslands Research Centre, Tennent Drive, Fitzherbert, 4410 Palmerston North, New Zealand; Shen.Hea@agresearch.co.nz
[5] Small Animal Colony, AgResearch Ltd., Ruakura Research Centre, 10 Bisley Road, Private Bag 3123, 3214 Hamilton, New Zealand; bobby.smith@agresearch.co.nz
* Correspondence: gosia.zobel@agresearch.co.nz; Tel.: +64-78385911

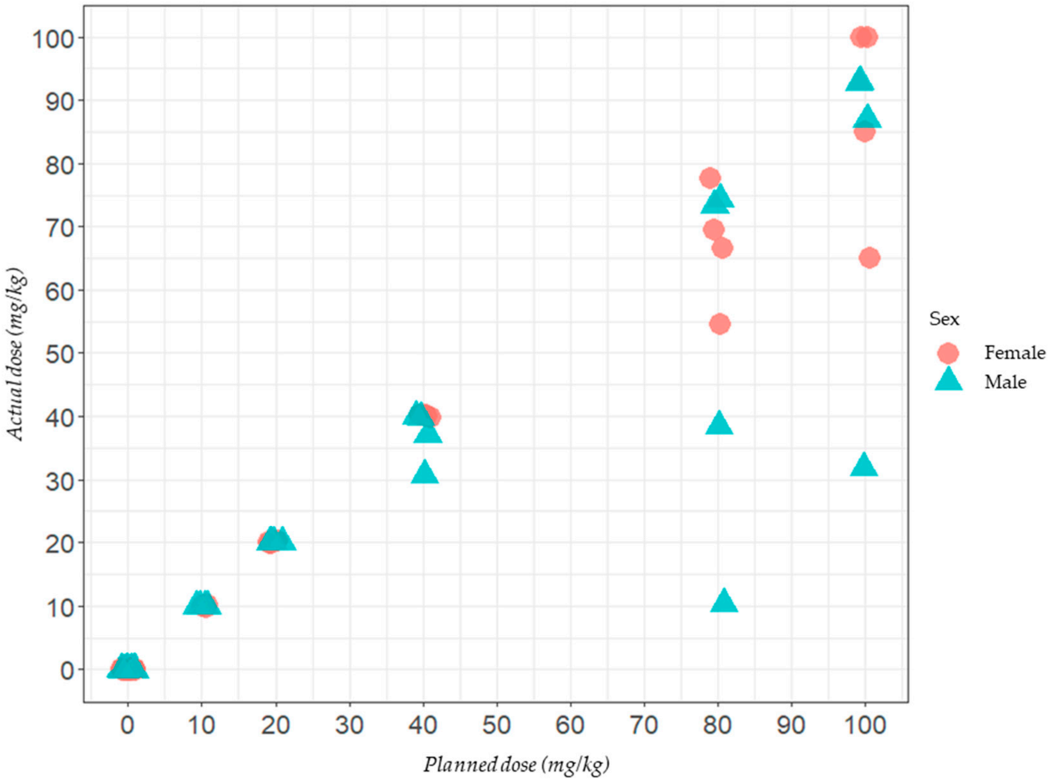

Figure S1. Planned *vs*. actual doses of tiletamine-zolazepam for each individual C57BL/6 male and female mouse. Planned doses were 0 (Control), 10 (Z10), 20 (Z20), 40 (Z40), 80 (Z80) and 100 (Z100) mg/kg, however, not all mice consumed the full amount of dispensed cream cheese on the day of euthanasia. Symbols have been jittered.

Table S1. Descriptive data for the behaviours recorded during the sedation period[1] in C57BL/6 mice.

Behaviour	Treatment	Mean	Min	Max	Q1	Median	Q3	SD	*n*[2]
Jumping[3] *(f)*									
	Control	0.2	0.0	5.0	0.0	0.0	0.0	0.88	38
	Z10	0.4	0.0	1.0	0.0	0.0	0.0	0.35	8
	Z20	5.6	0.0	30.0	0.0	0.0	4.3	10.98	8
	Z40	0.0	0.0	0.0	0.0	0.0	0.0	0.00	8
	Z80	0.0	0.0	0.0	0.0	0.0	0.0	0.00	8
	Z100	0.0	0.0	0.0	0.0	0.0	0.0	0.00	4
Rearing[3] *(f)*									
	Control	112.5	38.0	171.0	94.0	114.5	130.8	31.06	38
	Z10	117.9	80.0	156.0	97.8	119.5	134.3	28.40	8
	Z20	93.5	22.0	138.0	61.5	109.0	125.0	42.86	8
	Z40	73.5	1.0	131.0	36.5	71.0	122.5	48.91	8
	Z80	33.4	0.0	132.0	1.5	7.0	53.3	47.87	8
	Z100	24.8	8.0	50.0	12.5	20.5	32.8	18.61	4
Ataxia[4] *(s)*									
	Control	0.0	0.0	0.0	0.0	0.0	0.0	0.00	38
	Z10	0.4	0.0	1.5	0.0	0.0	0.6	0.56	8
	Z20	25.9	0.0	184.9	0.6	2.5	7.6	64.33	8
	Z40	62.6	0.7	393.7	1.3	5.1	31.9	136.25	8
	Z80	130.4	0.0	488.7	24.1	56.8	164.2	171.88	8
	Z100	245.2	4.8	515.4	12.7	230.3	462.8	273.04	4
Immobility[4] *(s)*									
	Control	0.1	0.0	3.4	0.0	0.0	0.0	0.55	38
	Z10	24.3	0.0	97.5	0.0	0.0	27.0	43.42	8
	Z20	40.4	0.0	153.5	0.0	17.0	50.4	56.92	8
	Z40	68.2	0.0	363.1	6.2	13.1	68.1	122.89	8
	Z80	64.3	0.0	199.4	4.4	28.1	118.7	76.38	8
	Z100	161.5	56.3	292.9	64.6	148.5	245.4	118.06	4
Laboured breathing[4] *(s)*									
	Control	0.0	0.0	0.0	0.0	0.0	0.0	0.00	38
	Z10	0.0	0.0	0.0	0.0	0.0	0.0	0.00	8
	Z20	0.0	0.0	0.0	0.0	0.0	0.0	0.00	8
	Z40	0.0	0.0	0.0	0.0	0.0	0.0	0.00	8
	Z80	14.6	0.0	117.0	0.0	0.0	0.0	41.37	8
	Z100	0.0	0.0	0.0	0.0	0.0	0.0	0.00	4

Table S1. Continued.

Behaviour	Treatment	Mean	Min	Max	Q1	Median	Q3	SD	*n*[2]
Recumbency[4] *(s)*									
	Control	0.0	0.0	0.0	0.0	0.0	0.0	0.00	38
	Z10	0.3	0.0	2.5	0.0	0.0	0.0	0.88	8
	Z20	8.9	0.0	64.6	0.0	0.0	1.6	22.63	8
	Z40	1.8	0.0	9.6	0.0	0.0	1.8	3.39	8
	Z80	342.9	0.0	786.4	0.0	308.4	627.8	357.72	8
	Z100	83.5	1.3	170.5	1.8	81.2	162.9	94.63	4
Walking on lid[4] *(s)*									
	Control	87.3	0.0	252.5	47.0	71.0	111.4	63.75	38
	Z10	24.9	0.0	47.4	11.3	24.2	39.6	17.82	8
	Z20	18.7	0.0	82.1	0.0	3.4	24.6	29.52	8
	Z40	27.4	0.0	135.3	0.0	6.7	30.3	46.51	8
	Z80	2.3	0.0	11.1	0.0	0.0	1.9	4.41	8
	Z100	0.0	0.0	0.0	0.0	0.0	0.0	0.00	4
Paddling[4] *(s)*									
	Control	0.0	0.0	0.0	0.0	0.0	0.0	0.00	38
	Z10	0.0	0.0	0.0	0.0	0.0	0.0	0.00	8
	Z20	0.0	0.0	0.0	0.0	0.0	0.0	0.00	8
	Z40	0.0	0.0	0.0	0.0	0.0	0.0	0.00	8
	Z80	23.8	0.0	169.7	0.0	0.0	7.4	59.22	8
	Z100	0.0	0.0	0.0	0.0	0.0	0.0	0.00	4
Shaking[4] *(s)*									
	Control	0.0	0.0	0.0	0.0	0.0	0.0	0.00	38
	Z10	7.8	0.0	62.7	0.0	0.0	0.0	22.17	8
	Z20	2.4	0.0	19.3	0.0	0.0	0.0	6.82	8
	Z40	1.9	0.0	11.9	0.0	0.0	0.9	4.22	8
	Z80	358.4	0.0	786.4	0.0	284.7	755.3	378.20	8
	Z100	14.0	0.0	43.9	0.0	6.0	20.0	20.74	4

[1] These behaviours were recorded for the first 800 seconds of the observation period.

[2] Each treatment contained 2 cages of female pair-housed mice and 2 cages of male pair-housed mice; each of these was matched with a randomly assigned control, resulting in a total of 8 control mice per treatment, and a total of 40 control mice for the entire study. There were 2 mice from Control group and 4 mice from Z100 treatment that could not be considered for the behavioural analysis during the sedation period due to a camera recording issue.

[3] Jumping and rearing were reported as number of incidents (*frequency, f*).

[4] Ataxia, immobility, laboured breathing, recumbency, walking on lid, paddling, and shaking were reported as durations (*seconds, s*).

Table S2. Descriptive data for the duration[1] of behaviours recorded during the euthanasia period in C57BL/6 mice

Behaviour	Treatment	Mean	Min	Max	Q1	Median	Q3	SD	n^2
Latency to ataxia from CO_2 started (s)									
	Control	16.0	10.5	20.8	14.7	16.2	17.2	2.36	40
	Z10	16.4	11.4	20.4	15.5	16.1	17.4	2.86	8
	Z20	16.6	13.3	18.7	16.2	16.6	17.3	1.68	8
	Z40	10.1	2.0	16.2	5.7	11.4	15.2	6.11	8
	Z80	11.4	8.3	14.5	9.9	11.3	12.7	2.61	4
	Z100	6.7	2.3	14.0	2.8	3.8	11.1	5.50	6
Latency to recumbency from ataxia (s)									
	Control	8.7	3.8	19.6	6.8	7.9	9.6	3.30	40
	Z10	6.9	4.9	10.5	5.6	6.2	7.4	2.19	8
	Z20	9.5	5.7	14.1	8.5	9.2	10.5	2.45	8
	Z40	14.8	9.2	19.9	11.8	15.0	18.3	4.19	8
	Z80	11.0	9.1	14.4	9.5	10.3	11.8	2.37	4
	Z100	14.6	4.5	20.9	9.5	17.4	19.7	7.07	6
Latency to laboured breathing from recumbency (s)[3]									
	Control	1.3	-0.5	4.2	0.7	0.9	1.6	0.92	40
	Z10	1.5	0.8	2.4	1.2	1.3	1.8	0.50	8
	Z20	3.1	0.6	8.6	1.8	2.7	3.5	2.46	8
	Z40	1.1	0.3	2.6	0.8	0.9	1.3	0.68	8
	Z80	0.5	-0.9	1.0	0.4	0.9	0.9	0.91	4
	Z100	1.8	0.6	3.0	0.9	1.7	2.7	1.06	6
Latency to last breath from laboured breathing (s)									
	Control	70.4	59.7	99.5	65.7	70.7	73.3	7.33	40
	Z10	71.1	57.7	86.9	62.3	71.6	78.3	10.70	8
	Z20	76.5	62.6	92.5	69.5	75.1	82.8	10.68	8
	Z40	80.8	72.6	100.1	76.0	79.0	82.3	8.78	8
	Z80	83.6	69.6	89.1	82.4	87.8	89.0	9.39	4
	Z100	91.8	83.0	106.2	84.0	90.4	96.8	9.43	6

[1] All behaviours reported as durations (*seconds, s*).

[2] Each treatment contained 2 cages of female pair-housed mice and 2 cages of male pair-housed mice; each of these was matched with a randomly assigned control, resulting in a total of 8 control mice per treatment, and a total of 40 control mice for the entire study. There were 4 mice in Z80 group and 2 mice in Z100 group that were already recumbent before the euthanasia and could not be considered for the progression of events during the euthanasia.

[3] Some mice had already become recumbent prior to experiencing laboured breathing, which resulted in negative values for this variable.
